# Safety and efficacy of stoma site selection in CT-guided percutaneous gastrostomy: a retrospective analysis

**DOI:** 10.1186/s12957-024-03323-7

**Published:** 2024-02-06

**Authors:** Hu Chang-ming, Qi Xiao-mei, Liu Li, Liang Qing-Hua, Xiong Jun-ru, Li Liang-shan, Deng Liang-yu, Huang Xue-quan, He Chuang

**Affiliations:** Department of Nuclear Medicine (Treatment Center of Minimally Invasive Interventional), First Affiliated Hospital of Army Medical University, No. 30 of Gao Tanyan District, Chongqing, China

**Keywords:** Gastrostomy, CT-guided, Intercostal, Rectus abdominis

## Abstract

**Purpose:**

To compare the safety and efficacy of CPG in the rectus abdominis and intercostal regions.

**Materials and methods:**

This retrospective study included 226 patients who underwent CPG at a single center, with the stoma placed in the rectus abdominis or intercostal region. Surgical outcomes and complications, such as pain and infection within 6 months postoperatively, were recorded.

**Results:**

The surgical success rate was 100%, and the all-cause mortality rate within 1 month was 0%. An intercostal stoma was placed in 56 patients; a rectus abdominis stoma was placed in 170 patients. The duration of surgery was longer for intercostal stoma placement (37.66 ± 14.63 min) than for rectus abdominis stoma placement (30.26 ± 12.40 min) (*P* = 0.000). At 1 month postsurgery, the rate of stoma infection was greater in the intercostal group (32.1%) than in the rectus abdominis group (20.6%), but the difference was not significant (*P* = 0.077). No significant difference was observed in the infection rate between the two groups at 3 or 6 months postsurgery (*P* > 0.05). Intercostal stoma patients reported higher pain scores during the perioperative period and at 1 month postsurgery (*P* = 0.000), but pain scores were similar between the two groups at 3 and 6 months postsurgery. The perioperative complication rates for intercostal and rectus abdominis surgery were 1.8% and 5.3%, respectively (*P* = 0.464), with no significant difference in the incidence of tube dislodgement (*P* = 0.514). Patient weight improved significantly at 3 and 6 months postoperatively compared to preoperatively (*P* < 0.05).

**Conclusion:**

Rectus abdominis and intercostal stomas have similar safety and efficacy. However, intercostal stomas may result in greater short-term patient discomfort.

## Introduction

Percutaneous endoscopic gastrostomy (PEG) is an efficacious treatment modality for patients with head and neck tumors, esophageal tumors, or other conditions that can lead to swallowing difficulties. However, obstacles during esophageal endoscopy may occur in certain patients. For patients who need artificial enteral nourishment, percutaneous radiological gastrostomy (PRG) is the favored technique. PRG is characterized by an exceptionally high technical success rate and a low incidence of severe complications [1, 2]. Moreover, for patients with swallowing difficulties due to esophageal obstruction, PRG has been shown to result in a better prognosis and fewer complications than metal stent implantation [3].

Research primarily comparing the clinical outcomes of PRG and PEG has been conducted. Although the success rates of these two methods are comparable, higher rates of tube leakage and tube dislodgement have been noted after PRG [4]. Despite no significant difference between the two procedures in terms of the rate of bleeding or infection around the stoma, PRG is associated with a higher rate of adverse events such as colon perforation and peritonitis [5, 6]. Currently, digital subtraction angiography (DSA), computed tomography (CT), and C-arm CT are the primary imaging modalities used to guide percutaneous gastrostomy, with DSA guidance being the most commonly reported. In 1992, successful CT-guided percutaneous gastrostomy (CPG) was performed in patients who could not undergo fluoroscopy for anatomical or pathological reasons [7]. CT offers high-density resolution, thereby enabling clear visualization of important structures such as blood vessels, the colon, and the liver. Enhanced, thin-slice CT can also distinctly display small arteries in the stoma area [8], and stoma site selection is more feasible with CPG. The preferred regions for stoma placement include the gastric body, gastric fundus, and upper region of the rectus abdominis; however, there have been few reports of intercostal stoma placement [9, 10].

In situations in which stoma creation in the rectus abdominis region is not feasible for objective reasons, exploring alternative options is necessary. To understand the relationship between stoma creation in different regions and the incidence of postoperative complications, we conducted a retrospective analysis of stoma site selections in the CPG, and the results provide helpful evidence for clinical decision-making regarding gastrostomy.

## Patients and methods

### Patients

The ethics committee of our institution approved this study of 226 patients who underwent CPG from 2019 to 2023. Patients signed informed consent forms before surgery; however, the need for informed consent was waived by the review board due to the anonymized, retrospective design of the study. Among the patients, 185 were male and 41 female, with an average age of 65.17 ± 10.47 years. Diagnoses included the following: esophageal tumor (173 patients), head and neck tumor (24), esophagobronchial fistula (5), amyotrophic lateral sclerosis (12), lung cancer with esophageal obstruction (7), swallowing dysfunction after cerebral infarction (2), and individual cases of hereditary ataxia, esophageal chemical burn, or severe traumatic brain injury causing swallowing dysfunction.

### Preoperative evaluation and equipment

Before the procedure, several critical evaluations were conducted, including electrocardiography and determination of the PLT level (≥ 50 × 10^9^/L), international normalized ratio (INR, corrected to 0.8–1.6), and activated partial thromboplastin time (APTT), which must not exceed the normal limit by more than onefold. Moreover, electrocardiography showed no evidence of acute myocardial ischemia. All anticoagulant and antiplatelet medication therapies were suspended before surgery, and heparin bridging therapy was adjusted according to the patient’s medication profile. Heparin was discontinued 24 h prior to surgery.

An enhanced CT scan was performed preoperatively to examine the gastrostomy area for vascular abnormalities such as varices. A UCT510 (United Imaging Healthcare Co., Ltd., China) system was used for CPG, with the following scanning parameters: tube voltage, 120 kV; tube current, 200 mA; and slice thickness, 1.5–5 mm. Multiplanar reconstruction (MPR) was carried out based on the intraoperative conditions.

Before surgery, sedation and analgesia were achieved through injection of 0.1 g of sodium phenobarbital (Min Dong Li-jie-Xun Pharmaceutical Co., Ltd., China) and 0.1 g of pentazocine (Northeast Pharmaceutical Group Shenyang First Pharmaceutical Co., Ltd., China), respectively. Local anesthesia was induced using 0.1 g of lidocaine hydrochloride (Tian-sheng Pharmaceutical Co., Ltd., China). The surgical area, including the rectus abdominis muscle and intercostal spaces 6–12, was disinfected with povidone-iodine solution (Hua-tian Technology Industrial Co., Ltd., China). The procedure was performed by experienced physicians skilled in interventional diagnosis and treatment under CT guidance.

### Principles for intercostal stoma placement

**Fig. 1:**
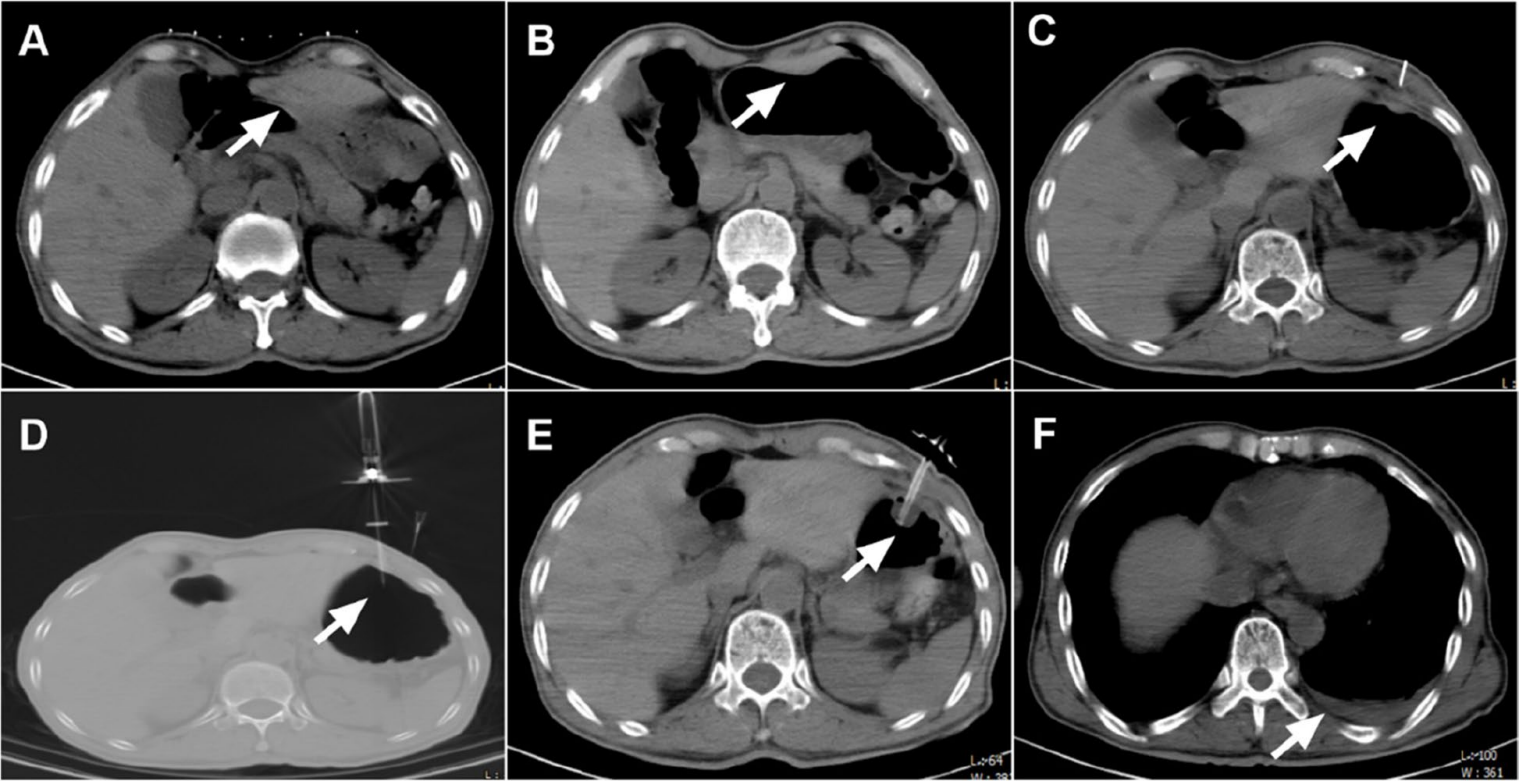
A 59-year-old male patient with esophageal squamous cell carcinoma and esophageal obstruction. Preoperative CT showed complete obstruction of the gastric cavity by the left hepatic lobe (**A**). Despite insufflation of the gastric cavity, the proposed site for rectus abdominis stoma placement was still obstructed by the left hepatic lobe (**B**). Therefore, the decision was made to place the stoma at the left 6th intercostal space (**C**). A 20-G gastric wall fixator was used to secure the gastric wall (**D**). Following wall fixation, a 16-Fr support sheath was inserted to facilitate fistula creation, and a 15-Fr gastric catheter was inserted through the opening (**E**). Postoperative CT revealed minimal bleeding in the left thoracic cavity (**F**)

The rectus abdominis area is preferred for stoma placement if there is no organ interference in either the intercostal or rectus abdominis areas after gastric insufflation. In cases in which the rectus abdominis area is obstructed by the liver or colon and cannot be circumvented, the option of intercostal stoma placement may be considered (Fig. 1A, B). The stoma should be placed in the intercostal area if there are skin lesions in the rectus abdominis area. However, the intercostal area can be considered for patients whose gastric anatomy is abnormal or for whom there are restrictions in the rectus abdominis area. The intercostal area should be chosen for stoma placement if the gastrostomy tube is near the liver or colon after gastric insufflation. The intercostal area may also be considered for stoma placement if prominent folds or depressions in the rectus abdominis area hinder local skin cleansing.

### Gastric insufflation process

Gastric insufflation was achieved using an 18 G soft tissue puncture needle and a 22-G sterile injection needle, avoiding the stoma area. Indications for successful puncture include withdrawal of gastric fluid and gas distribution analysis following the injection of 10–20 ml of gas. Once the gastric cavity was accessed, 300–500 ml of air was continuously injected, and the gastrostomy location was determined by the surrounding organ distribution after insufflation (Fig. 1B, C).

### Gastrostomy

After insufflation, a 20-G gastric wall fixator was inserted into the gastric cavity. CT was used to confirm the correct placement of the puncture needles for both wire holding and wire insertion. The stainless-steel ring of the former was opened to grasp the nylon thread inserted from the latter. Upon successful grasping, the fixator was removed, and the thread was tied to fix the gastric wall. This process was repeated 1–2 cm away from the initial fixation point (Fig. 1D). After fixation, CT was used to ensure proper fixation and gas filling. A 16 Fr support sheath was then used for fistula creation, and a 15 Fr gastric catheter was inserted through the opening. The distal end of the balloon was filled with 3 ml of sterile distilled water, and the catheter was then withdrawn, with any resistance prompting CT verification of the position of the balloon.

### Postgastrostomy

A CT scan was conducted to check for surgical complications and to confirm the position and inflation status of the balloon (Fig. 1E). The gastrostomy fixation plate was securely attached to the skin, with careful observation for any bleeding at the incision site. Dressing materials were applied to cover the stoma area. Moreover, efforts were made to remove all air and residual gastric fluid from the stomach, keeping the gastric cavity pressure low.

### Follow-up

In this study, the follow-up period was set at 6 months after CPG, during which specialized nursing staff provided care for the stoma site. Enteral feeding began at 24 h after the gastric tube was placed. After the patients were discharged, the nursing staff continued to provide care through a WeChat video communication platform. They monitored and guided each patient and their family in-home care, which included dressing changes, cleaning the stoma site, and daily maintenance of the gastric tube. They also diligently recorded any tube-related symptoms, signs, or complications observed during the follow-up process. The sutures used to fix the gastric wall were removed between 14 and 30 days after the CPG procedure. The first tube replacement procedure was performed between 3 and 6 months after CPG; the tube could be removed once the patient was able to tolerate oral intake.

### Definitions

Intercostal space: The space between adjacent ribs. Rectus abdominis: Abdominal muscles in the space between the lower rib cage and the umbilicus. Perioperative numerical rating scale (NRS) score: The highest score recorded after CPG. Perioperative period: The time span including preoperative, intraoperative, and postoperative care until patient discharge. Perioperative complications: Complications occurring during the perioperative period, including pleural and peritoneal reactions and bleeding events, are evaluated and recorded by the operating physician and nursing staff. Surgical duration: Time difference between when the first and last intraoperative images were obtained, including during gastric insufflation. Stoma infection: The presence of signs of infection around the stoma site, such as redness, swelling, pain, or pus. Tube dislodgement: Complete catheter dislodgement, with CT showing catheter dislodgement outside the gastric cavity or in the abdominal wall or cavity. Balloon rupture: as indicated by leakage during gastrostomy tube removal.

### Statistical analysis

All data are presented as means ± standard deviations for continuous variables and as numerical values (percentages) for categorical variables. Data were compared by univariate analysis using *t* tests or one-way ANOVA for continuous variables and Pearson’s *χ*2 test, likelihood ratio test, or Fisher’s exact test for categorical variables. All reported *P* values are two-sided and were not adjusted for multiple testing; *P* < 0.05 was considered to indicate a statistically significant difference. The statistical analysis was performed by using SPSS Statistics (version 26, IBM).

## Results

**Fig. 2:**
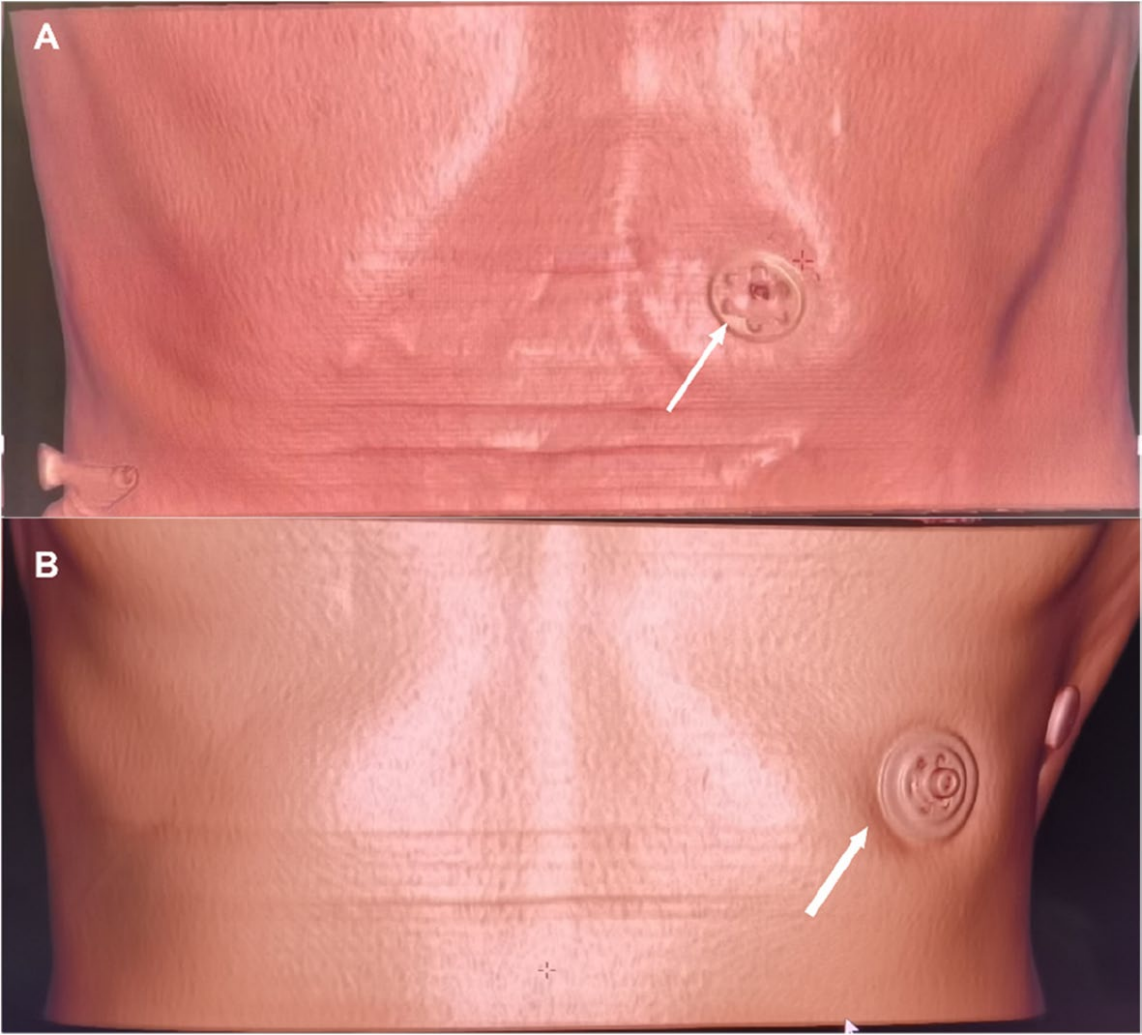
CT virtual reconstruction imaging results for two patients who underwent gastrostomy. **A** The site of gastrostomy located in the rectus abdominis region. **B** The site of gastrostomy located between the 7th and 8th ribs

There were 185 male and 41 female patients, with an average age of 65.17 ± 10.47 years. The surgery had a high overall success rate. Among the patients, 56 underwent stoma placement in the intercostal area (Fig. 2A), with 39.3%, 55.4%, and 5.4% of the lesions located in intercostal spaces 6, 7, and 8, respectively. The average duration of CPG was 37.66 ± 14.63 min, with a duration of gastric insufflation of 11.24 ± 9.91 min. Among the 170 patients who underwent stoma placement in the rectus abdominis area (Fig. 2B), the average duration of CPG was 30.26 ± 12.40 min, with a duration of gastric insufflation of 8.01 ± 5.59 min. There was a significant difference in the duration of CPG placement between patients with intercostal and rectus abdominis stomas (*t* = 3.696, *P* = 0.00).

During the perioperative period (Table 1), the NRS score was distributed as follows: 10 patients had a score of 0 (4.4%), 160 patients had a score between 1 and 3 (70.8%), and 56 patients had a score between 4 and 6 (24.8%). The median perioperative NRS score for patients with intercostal stomas was 4 (IQR 2, 5), whereas the median score for patients with rectus abdominis stomas was 2 (IQR 1, 2) (*Z* =  − 6.684, *P* < 0.0001). Within the first month after surgery, NRS scores were 3 (IQR 0, 4) and 0, respectively, with the pain scores of patients with stomas in the rectus abdominis region being significantly lower than those of patients with stomas in the intercostal region (*Z* =  − 5.593, *P* < 0.0001). NRS scores at 3 and 6 months postsurgery were comparable between the two groups. The overall stoma site infection rate 1 month postsurgery was 23.4%. The infection rate was 32.1% (18/56) for stomas in the intercostal region and 20.6% (35/170) for stomas in the rectus abdominis region, revealing a significant difference between the two regions (*Z* = 3.133, *P* = 0.077). However, there was no significant difference in infection rate between the rectus abdominis and intercostal regions at 3 or 6 months after surgery (*P* > 0.05).

**Table 1:** Clinical data, surgical results, and complications in CPG patients

	Intercostal	Rectus abdominis	*P*
Sex			0.053
Male	41	144	
Female	15	26	
Age	62.55 ± 11.02	66.03 ± 10.17	0.031
Clinical diagnoses			0.619
Esophageal tumor	46	127	
HNT	5	19	
Esophagobronchial fistula	0	5	
ALS	3	9	
LCEO	2	5	
Hereditary ataxia	0	1	
ECB	0	1	
Cerebral infarction	0	2	
STBI	0	1	
CPG time	37.66 ± 14.63	20.26 ± 12.40	0.000
Gastric insufflation time	11.24 ± 9.71	8.01 ± 5.59	0.004
NRS			
Perioperative	4(IQR 2,5)	2(IQR 1,2)	0.000
1 month	3(IQR 0,4)	0	0.000
3 months	0	0	0.299
6 months	0	0	0.564
Stoma infection			
(1 month) yes	18	35	0.077
(1 month)no	38	135	
(3 months) yes	4	8	0.816
(3 months) no	52	149	
(6 months) yes	3	4	0.505
(6 months) no	47	146	
Perioperative complications			0.464
Yes	1	9	
No	55	170	
Dislodgement			0.514
Yes	11	27	
No	45	143	

*CPG* CT-guided percutaneous gastrostomy, *HNY* head and neck tumor, *ALS* amyotrophic lateral sclerosis, *LCEO* lung cancer with esophageal obstruction, *ECB* esophageal chemical burn, *STBI* severe traumatic brain injury, *IQR* interquartile range

There were 3 cases each of subcutaneous hematoma, intra-abdominal bleeding, and rectus abdominis hematoma (which included one case of bleeding and hematemesis on day two post-CPG) and one case of thoracic bleeding (Fig. 1F). The perioperative complication rate was 5.3% (9/170) for stomas in the rectus abdominis region and 1.8% (1/56) for stomas in the intercostal region, with no significant difference in the complication rate between the two regions (*Z* = 0.537, *P* = 0.464). One patient with a rectus abdominis stoma experienced bleeding from the surgical site on the fifth day after discharge, which ceased after symptomatic treatment. No patients experienced peritoneal or pleural reactions during the perioperative period.

Most incidences of tube dislodgement occurred on the 42nd day (IQR 22–101) after initial tube placement. The rate of tube dislodgement was 19.6% for stomas in the intercostal region and 15.9% for those in the rectus abdominis region, with no significant difference (*χ*2 = 4.26, *P* = 0.514).

This study showed that complications related to CPG are not typically fatal. The all-cause mortality rate was 11.5% during the follow-up period, but none of these deaths occurred within the first month following CPG. Most deaths occurred in patients with a rectus abdominis stoma, with mortality rates of 5.7% and 4.5% at 3 and 6 months postsurgery, respectively. Moreover, the 6-month mortality rate was slightly greater in patients with stomas in the intercostal region (10.7%). Nonetheless, there was no significant difference in the all-cause mortality rate between patients with a stoma in the rectus abdominis or intercostal region (*Z* = 2.819, *P* = 0.093). Moreover, surviving patients showed a significant improvement in weight at 3 and 6 months following CPG (*P* < 0.005).

## Discussion

To ensure intestinal nutrition, PEG is a common treatment method for patients who cannot tolerate oral intake. However, some patients cannot tolerate PEG. PRG is a safe and effective alternative that involves direct percutaneous puncture of the stomach and is recommended for use in guidelines [11, 12]. CT has high predictive value for determining the success of percutaneous gastrostomy [13]. CPG is an important treatment method that can be used as a supplementary approach when gastrostomy tubes cannot be placed through endoscopy or fluoroscopy [14]. This procedure has a high success rate ranging from 97.7 to 100% [7, 15], with no cases of fistula implantation metastasis, as reported by Ellrichmann [16].

In gastrostomy, the fistula is predominantly situated in the gastric body or fundus; leakage from the opening in the abdominal wall occurs primarily in the superior rectus abdominis region. Reports of stoma placement in the intercostal region are rare [9, 10]. Notably, surgical interventions involving stoma placement in the 7th and 8th intercostal spaces have demonstrated promising clinical results [17]. These isolated findings suggest the efficacy of an intercostal stoma as an alternative treatment. Therefore, informed by real-time gastric insufflation and considering proximity to crucial neighboring organs and the skin, our chosen site for stoma placement was within anterior intercostal spaces 6 to 8.

Although the overall incidence of stoma infection in our study was slightly lower than that reported by Krishna et al. [18], the overall degree of pain during the perioperative period was slightly greater than that reported in a study by Philip et al. [19]. This increase in pain was primarily noted within the first month following CPG. The results showed that a stoma in the intercostal region was associated with a greater level of pain and risk of infection than a stoma in the rectus abdominis region. This increase in discomfort may be attributed to irritation caused by respiratory movements or tugging of the intercostal nerves. Such discomfort may deter patients from adhering to consistent comprehensive cleaning and care of the stoma. Moreover, patients with less subcutaneous fat may have anatomical depressions in the intercostal area, inhibiting thorough disinfection of the stoma site and subsequently augmenting the risk of infection. However, there was no significant difference in the incidence of such minor complications at 3 or 6 months. Consequently, we posit that intercostal stoma placement is a highly effective and significant method because minor complications are easily managed in the short term to mitigate patient discomfort. With a thorough understanding of these factors, tailored postoperative care plans can be designed for patients not suitable for stoma placement in the rectus abdominis region.

Bleeding after gastrostomy is difficult to avoid, and reports suggest that approximately 1.4–2.5% of patients may experience injury to the inferior epigastric artery, resulting in iatrogenic pseudoaneurysm, rectus sheath hematoma, and wound oozing [20, 21]. Although preoperative thin-slice enhanced CT can reveal the course of blood vessels in the rectus abdominis and intercostal areas [8], the incidence of bleeding in this study was slightly greater than that reported by Yasin et al. [15]; however, the bleeding volume was lower, and all cases of bleeding were successfully managed conservatively. In the study of Yasin et al., 6% of patients underwent interventional embolization or surgical intervention. Furthermore, intercostal gastrostomy may cause damage to intercostal blood vessels, leading to thoracic bleeding. One such case was observed in the present study, but the bleeding was stopped by fixing the gastric wall with local sutures and applying pressure using a catheter. Therefore, in intercostal gastrostomy, caution should be exercised when dealing with intercostal blood vessels to avoid injury.

Tube dislodgement after percutaneous gastrostomy can be caused by various factors. Several studies suggest that the rate of tube dislodgement is greater with the PRG approach [4]. However, our study revealed that the overall risk of dislodgement was lower than that reported by Vidhya [22], who reported similar risks between the rectus abdominis and intercostal regions. Therefore, postoperative continuous care management should be strengthened to reduce the risk of tube-related issues caused by misoperation at home.

By standardizing the surgical procedure, shorter surgical durations may be achieved. Although the overall surgical duration for both the rectus abdominis and intercostal regions was slightly longer than the 23.8 ± 1.39 min reported for PEG by Zhang et al. [23], the duration of gastric insufflation in our study ranged from 8 to 11 min, which clearly exceeded the values reported by Zhang et al. [24]. Since the surgical duration is one of the factors associated with aspiration after tube implantation [25], further improvements in gastric insufflation methods are needed to shorten the surgery duration. Furthermore, regardless of whether the CPG stoma is located in the rectus abdominis or intercostal region, it serves to deliver nutrients, as in PEG [5], suggesting positive recovery trajectories for the majority of patients.

## Limitations

This retrospective data analysis has certain limitations. First, regarding the selection of the gastrostomy site, it is not possible to conduct a prospective randomized study. In future studies, it may be worth clarifying the criteria for selecting a gastrostomy site via either the rectus abdominis or intercostal route. Second, the follow-up period of 6 months may be insufficient for a comprehensive evaluation of long-term complications. Additionally, our analysis of complications did not include the occurrence of pneumoperitoneum. During data compilation, gastric insufflation often resulted in minimal pneumoperitoneum, which may have impacted the analysis. Furthermore, the small amount of pneumoperitoneum was not given specific attention or treatment in this study. Moreover, sterile gas was not utilized for gastric insufflation, with the possibility of infection. Finally, the radiation dose received by the patients in the two groups was not evaluated.

## Conclusion

The use of an intercostal stoma in CPG as a substitute for a rectus abdominis stoma is regarded as a safe and effective method. It is important to improve postoperative care to reduce the chances of tube dislodgement. Additionally, establishing efficient communication channels and promptly addressing any minor short-term complications in patients with an intercostal stoma is crucial.

## Data Availability

The data supporting the findings of this study are available from the corresponding author upon reasonable request. The corresponding author, [He Chuang], can be contacted at [Email Address: longtoo123@qq.com] to request access to the data. We did not share the data publicly due to privacy concerns related to sensitive participant information.
